# Metformin Use Is Associated With a Lower Incidence of Hospitalization for Atrial Fibrillation in Patients With Type 2 Diabetes Mellitus

**DOI:** 10.3389/fmed.2020.592901

**Published:** 2021-02-22

**Authors:** Chin-Hsiao Tseng

**Affiliations:** ^1^Department of Internal Medicine, National Taiwan University College of Medicine, Taipei, Taiwan; ^2^Division of Endocrinology and Metabolism, Department of Internal Medicine, National Taiwan University Hospital, Taipei, Taiwan; ^3^Division of Environmental Health and Occupational Medicine of the National Health Research Institutes, Zhunan, Taiwan

**Keywords:** atrial fibrillation, diabetes mellitus, metformin, Taiwan, National Health Insurance

## Abstract

**Background:** The effect of metformin on the risk of atrial fibrillation (AF) requires confirmation. This retrospective cohort study compared the incidence of hospitalization for AF in ever and never users of metformin.

**Methods:** Patients with newly diagnosed type 2 diabetes mellitus during 1999–2005 were enrolled from Taiwan's National Health Insurance database. Analyses were conducted in both an unmatched cohort of 173,398 ever users and 21,666 never users and in a propensity score-matched cohort of 21,662 pairs of ever and never users. They were free from a diagnosis of AF before January 1, 2006 and were followed up until December 31, 2011. Hazard ratios were estimated by Cox regression incorporated with the inverse probability of treatment weighting using the propensity score.

**Results:** A total of 303 ever users and 86 never users in the unmatched cohort and 56 ever users and 86 never users in the matched cohort developed hospitalization for AF during follow-up. The respective incidence rates were 37.72 and 92.45 per 100,000 person-years in the unmatched cohort and were 56.98 and 92.46 per 100,000 person-years in the matched cohort. The hazard ratio for ever vs. never users was 0.405 (95% confidence interval: 0.319–0.515) in the unmatched cohort and 0.617 (0.441–0.864) in the matched cohort. Hazard ratios for the tertiles of cumulative duration of metformin therapy vs. never users showed a dose-response effect. The findings were consistent in sensitivity analyses.

**Conclusion:** Metformin use is associated with a lower risk of hospitalization for AF in patients with type 2 diabetes mellitus.

## Introduction

Atrial fibrillation (AF) is a common arrhythmia that can lead to increased hospitalization, stroke, or even life-threatening thromboembolic events (1, 2). Risk factors of AF may include aging, male sex, smoking, alcohol consumption, diabetes mellitus, hypertension, obesity, thyroid dysfunction, obstructive sleep apnea, high-level physical training, left ventricular dysfunction, valvular heart disease, myocardial infarction, and heart failure (2–4). The estimated lifetime risk of AF is 22–26% and diabetes patients suffer from a 1.4–1.6-fold higher risk (2, 3). While compared to diabetes patients without AF, diabetes patients with AF have a 61% higher risk of total mortality, 77% higher risk of cardiovascular death, and 68% higher risk of heart failure (5).

Metformin, now a first-line oral antidiabetic drug recommended for the treatment of type 2 diabetes mellitus, exerts an insulin sensitizing effect (6), and may have anti-inflammatory, anti-aging, anti-cancer, and even anti-microbial effects (7–10). Two previous pharmacoepidemiological studies conducted in Taiwan that used the nationwide reimbursement database of the National Health Insurance (NHI) showed that patients with type 2 diabetes mellitus treated with metformin might have a 20% lower risk of AF (11, 12). However, these earlier studies suffered from some methodological limitations.

There are several methodological limitations in the study by Chang et al. (11). First, the metformin users and non-users enrolled were highly imbalanced in the use of statin with a significantly higher rate of statin use among metformin users. Because statin may reduce the incidence of AF by 40–50% (13, 14), the lower risk of AF among incident users of metformin could be potentially ascribed to the effect of statin which had been in use for a certain period before metformin was initiated. Second, the significantly higher prevalence rates of important risk factors of AF among non-users of metformin, such as male patients, older age, congestive heart failure, chronic kidney disease, asthma, hyperthyroidism, myocardial infarction, ischemic stroke and peripheral arterial disease, might have exerted residual confounding effects even though some of them were considered in the adjusted model. Third, imbalance in the use of other antidiabetic drugs between metformin users and non-users was highly probable and this might have led to a biased result. The investigators excluded, at the beginning of the study, users of other antidiabetic drugs, and compared the incident cases of AF between metformin users and non-users over time by using a time-dependent approach. In this sense metformin non-users represented a highly selective group of patients who had a diagnosis of diabetes mellitus but had not been treated with any other antidiabetic drugs at the beginning of the study. During subsequent years of follow-up, non-users of metformin might have been prescribed metformin or other antidiabetic drugs. Once metformin was prescribed in the subsequent years, the patients would be reclassified as metformin users. It was expected that insulin or insulin-secreting drugs, which could cause hypoglycemia and thus AF, would have been prescribed to metformin non-users for the purpose of glycemic control in the subsequent years of follow-up. Therefore, the proportions of the use of other antidiabetic drugs that could cause hypoglycemia and AF would be much higher among metformin non-users during any subsequent year of follow-up. Finally, this study did not consider the requirement of a sufficient induction time and did not address the potential bias due to immortal time. Therefore, selection bias, confounding by indication, and immortal time bias could not be excluded in this study.

In the study by Liou et al., a nested case-control design was used by including 11,528 diabetes patients without AF and 2,882 diabetes patients with AF (12). The investigators cross-sectionally estimated the odds ratios of AF for metformin users vs. metformin non-users (12). The cross-sectional design did not assure a temporal correctness of a cause (metformin use) that should happen before an effect (AF incidence). It is interesting that although a matching algorithm was used, the enrolled cases (AF group) and controls (non-AF group) were not well-matched in most of the important confounders. For example, the AF group was characterized by significantly higher prevalent rates of potential risk factors of AF including hypertension, congestive heart failure, chronic kidney disease, acute myocardial infarction, and ischemic stroke than the non-AF group. Furthermore, the enrolled AF group was characterized by significantly higher prevalence rates of the use of insulin, sulfonylurea, glinide, alpha-glucosidase inhibitors, and dipeptidyl-peptidase 4 inhibitors (12). Because insulin and insulin-secreting drugs may induce hypoglycemia which would lead to AF, residual confounding from other antidiabetic drugs could not be excluded. Therefore, this study could not clarify the temporal correctness of a cause-effect relationship and might have suffered from selection bias, prevalent user bias, immortal time bias, and confounding by indication.

Some other common limitations found in these two earlier studies (11, 12) included a lack of investigating a dose-response effect and an ignorance of the potential impact of the regularity of metformin use. Additionally, the diagnosis of AF was made mainly at outpatient settings in these two studies, which was prone to be misclassified than a diagnosis made at the discharge of a hospitalization that is always supported by laboratory tests performed during admission.

A recent study conducted in the USA showed an increasing trend of emergency department visits and hospitalization for AF (HAF) from 2007 to 2014 in a nationwide level and called for a “need for widespread implementation of effective strategies aimed at improving the management of patients with AF to reduce hospital admissions and the economic burden of AF” (15). Because metformin is considered the first-line antidiabetic drug for patients with type 2 diabetes mellitus, whether the use of metformin can prevent the potentially fatal disease of AF in these high-risk diabetes patients is of immense clinical importance.

The purpose of the present study was to clarify whether metformin could reduce the incidence of AF made at the discharge of a hospitalization by using the Taiwan's NHI reimbursement database. The potential methodological limitations seen in the previous studies (11, 12) were especially considered.

## Materials and Methods

The NHI has been implemented in Taiwan since March 1995. It is a unique and universal health care system with a high coverage rate of >99% of the Taiwan's population. The Bureau of the NHI has contracts with all hospitals and with 93% of all medical settings. The reimbursement database of the NHI kept computerized records of disease diagnoses, medication prescriptions, and clinical procedures and can be used for academic research if approved after ethics review. The present study was approved number 99274 by the Ethics Committee of the National Health Research Institutes. According to local regulations, the National Health Research Institutes de-identified the individuals in the database for the protection of privacy and the Ethics Committee approved the analyses of the database without the requirement of obtaining informed consent from the participants.

Detailed description of the database has been reported in a previously published paper (16). During the study period, the International Classification of Diseases, Ninth Revision, Clinical Modification (ICD-9-CM) was used for disease diagnoses; and diabetes mellitus was coded as 250.XX and AF as 427.31.

Non-random observational studies tend to be biased by confounding by indication because treatment allocation may be associated with baseline characteristics. Methodological approaches by using PS have been recommended to reduce such a potential risk. PS is the probability of treatment assignment estimated by a set of baseline characteristics and it is always calculated by logistic regression. PS can be used for matching in selecting an exposed group and a non-exposed group for study or can be used for stratification or adjustment in analyses (17). Additionally, Cox proportional hazards model can be incorporated with the inverse probability of treatment weighting (IPTW) using the PS (17). Austin recommended the use of matching on PS for cohort selection and the IPTW method for analyses (17). These two approaches were applied in the present study.

**Figure 1:**
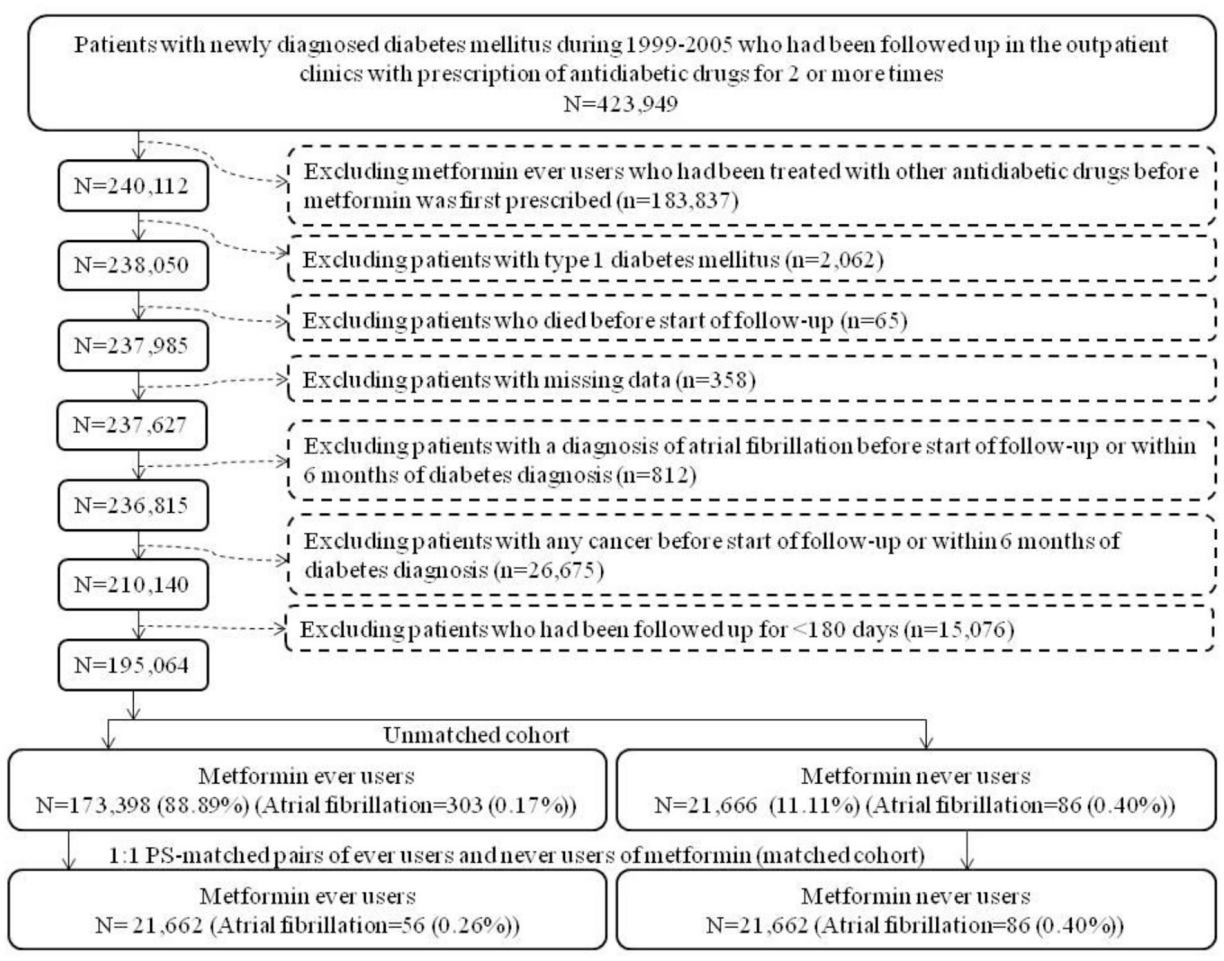
Flowchart showing the procedures in creating an unmatched cohort and a cohort of 1:1 propensity score-matched-pairs of metformin ever users and never users derived from the reimbursement database of the Taiwan's National Health Insurance (PS, propensity score).

The unmatched cohort and the PS-matched cohort were enrolled according to the procedures shown in Figure 1. At first, 423,949 patients who were newly diagnosed of diabetes mellitus during 1999–2005 in the outpatient clinics and had been prescribed antidiabetic drugs for two or more times were identified. The following ineligible patients were then excluded: (1) metformin ever users who had been treated with other antidiabetic drugs before metformin was initiated (*n* = 183,837); (2) type 1 diabetes mellitus (*n* = 2,062), (3) patients who died before the start of follow-up (*n* = 65); (4) missing data (*n* = 358), (5) diagnosis of AF before the start of follow-up or within 6 months of diabetes diagnosis (*n* = 812), (6) diagnosis of any cancer before the start of follow-up or within 6 months of diabetes diagnosis (*n* = 26,675), and (7) follow-up duration <180 days (*n* = 15,076). As a result, 173,398 ever users and 21,666 never users of metformin were identified as the unmatched cohort. PS was created from all characteristics listed in Table 1 plus the date of enrollment by logistic regression. A cohort of PS-matched pairs of 21,662 ever users and 21,662 never users (the matched cohort) was then created from the unmatched cohort by matching the PS using the Greedy 8 –>1 digit match algorithm proposed by Parsons (18).

**Table 1:** Characteristics in never users and ever users of metformin in the unmatched and matched cohorts.

	**Unmatched cohort**					**Matched cohort**				
**Variable**	**Never users**		**Ever users**			**Never users**		**Ever users**		
	**(*****n*** ***=*** **21666)**		**(*****n*** ***=*** **173398)**		**Standardized difference**	**(*****n*** ***=*** **21662)**		**(*****n*** ***=*** **21662)**		**Standardized difference**
	***n***	**%**	***n***	**%**		***n***	**%**	***n***	**%**	
**Demographic data**										
Age^*^ (years)	68.77	13.25	64.23	11.98	−41.47	68.76	13.25	68.70	12.09	0.18
Sex (men)	11,793	54.43	91,570	52.81	−3.47	11,790	54.43	11,731	54.15	−0.68
**Occupation**										
I	7,825	36.12	64,789	37.36		7,824	36.12	7,930	36.61	
II	3,646	16.83	36,710	21.17	12.61	3,646	16.83	3,634	16.78	−0.29
III	5,216	24.07	39,817	22.96	−2.67	5,215	24.07	5,187	23.95	−0.10
IV	4,979	22.98	32,082	18.50	−12.97	4,977	22.98	4,911	22.67	−0.69
**Living region**										
Taipei	7,328	33.82	54,416	31.38		7,325	33.81	7,279	33.60	
Northern	2,310	10.66	20,016	11.54	2.87	2,309	10.66	2,280	10.53	−0.48
Central	3,769	17.40	31,678	18.27	2.12	3,769	17.40	3,770	17.40	0.01
Southern	3,729	17.21	29,840	17.21	0.37	3,729	17.21	3,797	17.53	1.02
Kao–Ping and Eastern	4,530	20.91	37,448	21.60	2.59	4,530	20.91	4,536	20.94	0.22
**Major comorbidities**										
Hypertension	18,476	85.28	144,909	83.57	−5.99	18,473	85.28	18,528	85.53	0.98
Dyslipidemia	15,127	69.82	141,918	81.85	32.61	15,127	69.83	15,181	70.08	0.67
Obesity	518	2.39	7,364	4.25	11.02	518	2.39	485	2.24	−1.04
**Diabetes–related complications**										
Nephropathy	8,376	38.66	50,614	29.19	−24.85	8,373	38.65	8,280	38.22	−1.36
Eye diseases	3,741	17.27	55,119	31.79	35.93	3,741	17.27	3,646	16.83	−1.65
Hemorrhagic stroke	1,670	7.71	8,229	4.75	−15.04	1,670	7.71	1,604	7.40	−1.17
Non–hemorrhagic stroke	6,249	28.84	41,367	23.86	−14.07	6,248	28.84	6,286	29.02	0.47
Other types of stroke	6,512	30.06	40,938	23.61	−17.92	6,510	30.05	6,483	29.93	−0.08
Ischemic heart disease	11,689	53.95	84,499	48.73	−12.66	11,688	53.96	11,703	54.03	0.29
Peripheral arterial disease	5,678	26.21	47,465	27.37	2.04	5,676	26.20	5,648	26.07	−0.38
**Antidiabetic drugs**										
Insulin	1,891	8.73	3,953	2.28	−35.13	1,890	8.72	1,641	7.58	−6.31
Sulfonylurea	15,450	71.31	123,550	71.25	9.13	15,449	71.32	16,096	74.31	6.22
Meglitinide	1,958	9.04	6,930	4.00	−22.90	1,956	9.03	1,902	8.78	−1.17
Acarbose	2,443	11.28	9,223	5.32	−20.88	2,441	11.27	2,606	12.03	0.56
Rosiglitazone	618	2.85	8,158	4.70	10.82	618	2.85	615	2.84	−0.99
Pioglitazone	510	2.35	4,356	2.51	2.45	510	2.35	516	2.38	−0.67
**Commonly encountered comorbidities and potential risk factors of atrial fibrillation**										
Chronic obstructive pulmonary disease	12,054	55.64	89,210	51.45	−11.07	12,052	55.64	11,977	55.29	−0.56
Tobacco abuse	507	2.34	6,439	3.71	8.85	507	2.34	490	2.26	−0.54
Alcohol–related diagnoses	1,406	6.49	11,443	6.60	−0.12	1,406	6.49	1,300	6.00	−2.27
Cancer	2,254	10.40	13,711	7.91	−9.65	2,253	10.40	2,207	10.19	−0.68
Heart failure	6,237	28.79	35,109	20.25	−24.33	6,235	28.78	6,033	27.85	−2.13
Gout	8,921	41.18	63,552	36.65	−10.69	8,919	41.17	8,908	41.12	0.08
Hyperthyroidism	1,030	4.75	8,637	4.98	1.28	1,030	4.75	1,055	4.87	0.58
Sleep apnea syndrome	413	1.91	3,378	1.95	0.42	413	1.91	401	1.85	−0.41
Valvular heart disease	3,233	14.92	18,333	10.57	−16.23	3,233	14.92	3,111	14.36	−1.60
**Commonly used medications in diabetes patients**										
Angiotensin-converting enzyme inhibitors/angiotensin receptor blockers	15,959	73.66	130,207	75.09	2.71	15,957	73.66	15,880	73.31	−0.76
Calcium channel blockers	14,776	68.20	109,003	62.86	−12.95	14,773	68.20	14,794	68.29	0.47
Beta–blockers	15,951	73.62	119,761	69.07	−11.19	15,948	73.62	15,983	73.78	0.59
Statin	11,326	52.28	112,600	64.94	29.08	11,326	52.29	11,274	52.05	−0.56
Fibrate	7,090	32.72	73,563	42.42	22.48	7,090	32.73	7,013	32.37	−0.69
Aspirin	13,582	62.69	111,115	64.08	1.90	13,581	62.70	13,619	62.87	0.54

* *Age is expressed as mean and standard deviation*. *Refer to “Materials and Methods” for the classification of occupation*.

The start of follow up was set on January 1, 2006 and all comorbidities and covariates were determined as a status/diagnosis at any time before the start of follow-up. Potential confounders included: (1) demographic data: age, sex, occupation, and living region; (2) major comorbidities: hypertension (401-405), dyslipidemia (272.0-272.4), and obesity (278); (3) diabetes-related complications: nephropathy (580-589), eye diseases (250.5: diabetes with ophthalmic manifestations, 362.0: diabetic retinopathy, 369: blindness and low vision, 366.41: diabetic cataract, and 365.44: glaucoma associated with systemic syndromes), hemorrhagic stroke (430-432), non-hemorrhagic stroke (433-435), other types of stroke (436-438), ischemic heart disease (410-414), and peripheral arterial disease (250.7, 785.4, 443.81, and 440-448); (4) antidiabetic drugs: insulin, sulfonylurea, meglitinide, acarbose, rosiglitazone, and pioglitazone (rosiglitazone and pioglitazone are both thiazolidinediones but they may have different cardiovascular effects and thus were included separately); (5) commonly encountered comorbidities and potential risk factors of AF: chronic obstructive pulmonary disease (a surrogate for smoking, 490-496), tobacco abuse (305.1, 649.0 and 989.84), alcohol-related diagnoses (291, 303, 535.3, 571.0-571.3 and 980.0), cancer (140-208), heart failure (398.91, 402.11, 402.91, 404.11, 404.13, 404.91, 404.93, and 428), gout (274), hyperthyroidism (242), sleep apnea syndrome (327.2, 780.51, 780.53, and 780.57) and valvular heart disease (394-396, 424, and 746); and (6) commonly used medications in diabetes patients: angiotensin-converting enzyme inhibitors/angiotensin receptor blockers, calcium channel blockers, beta-blockers, statins, fibrates, and aspirin. The accuracy of disease diagnoses in the NHI database has been studied previously. Agreements between claim data and medical records are moderate to substantial, with Kappa values range from 0.55 to 0.86 (19).

The classifications of living region and occupation were detailed elsewhere (20). In brief, the living region was classified as Taipei, Northern, Central, Southern, and Kao-Ping/Eastern. Occupation was classified as class I (civil servants, teachers, employees of governmental or private businesses, professionals, and technicians), class II (people without a specific employer, self-employed people or seamen), class III (farmers or fishermen), and class IV (low-income families supported by social welfare, or veterans).

Standardized difference was calculated according to the methods proposed by Austin and Stuart for each covariate as a test of balance diagnostic (21). A value of >10% was used as a cutoff for potential confounding from the variable.

Cumulative duration of metformin therapy in months was calculated and its tertiles were used for dose-response analyses. Incidence density of HAF was calculated for never users, ever users, and users categorized according to the tertiles of cumulative duration of metformin therapy. The numerator of the incidence was the case number of newly diagnosed HAF as a primary diagnosis at the discharge of a hospitalization observed during follow-up. The denominator expressed in person-years was the follow-up time since January 1, 2006 until December 31, 2011, when a new diagnosis of HAF was made, or on the date of death or the last reimbursement record, whichever occurred first.

Kaplan-Meier curves for HAF-free probability were plotted for never users and ever users of metformin and for never users and users categorized according to the tertiles of cumulative duration of metformin therapy in the unmatched cohort and the matched cohort, respectively. Logrank test was used to test the significance in different subgroups of metformin exposure.

In main analyses, hazard ratios and their 95% confidence intervals were estimated by the IPTW method (17) in the unmatched cohort and the matched cohort, respectively. Models were created for ever users vs. never users and for users in each tertile of cumulative duration of metformin therapy in comparison to never users.

For sensitivity analyses, the following models were created in the unmatched cohort by using the IPTW method. At first, we excluded patients with irregular refill of metformin, based on two consecutive prescriptions of metformin spanning a period of >4 months (Model I, the Bureau of NHI allows a maximum of 3 months at each time of drug prescription for chronic diseases and these patients with delayed refill of metformin for more than 1 month after a previous 3-month prescription might represent those with poor adherence). We then excluded patients who happened to be treated with incretin-based therapies, i.e., dipeptidyl peptidase 4 inhibitors and glucagon-like peptide 1 receptor agonists, during follow-up (Model II, incretin-based therapies were not reimbursed by the Bureau of NHI in Taiwan until after 2009 and this analysis was aimed at excluding their potential impact). Because metformin may be contraindicated in patients with nephropathy or chronic kidney disease and an early study suggested that chronic kidney disease is associated with the incidence of AF in a dose-response pattern (22), additional analyses were conducted in patients with nephropathy (Model III), in patients without nephropathy (Model IV), after excluding patients with a diagnosis of chronic kidney disease (ICD-9-CM 585, Model V), after excluding patients in a renal dialysis status (ICD-9-CM V45.1, Model VI) and after excluding patients with a diagnosis of chronic kidney disease and/or in a renal dialysis status (Model VII).

Analyses were conducted using SAS statistical software, version 9.4 (SAS Institute, Cary, NC). *P* < 0.05 was considered statistically significant.

## Results

Table 1 shows the characteristics in never users and ever users of metformin in the unmatched cohort and the matched cohort, respectively. Before matching, ever users and never users showed imbalanced distribution of many covariates with standardized difference >10%. However, after matching, the two groups were well-balanced in all covariates and none of the variables had a value of standardized difference >10%.

**Figure 2:**
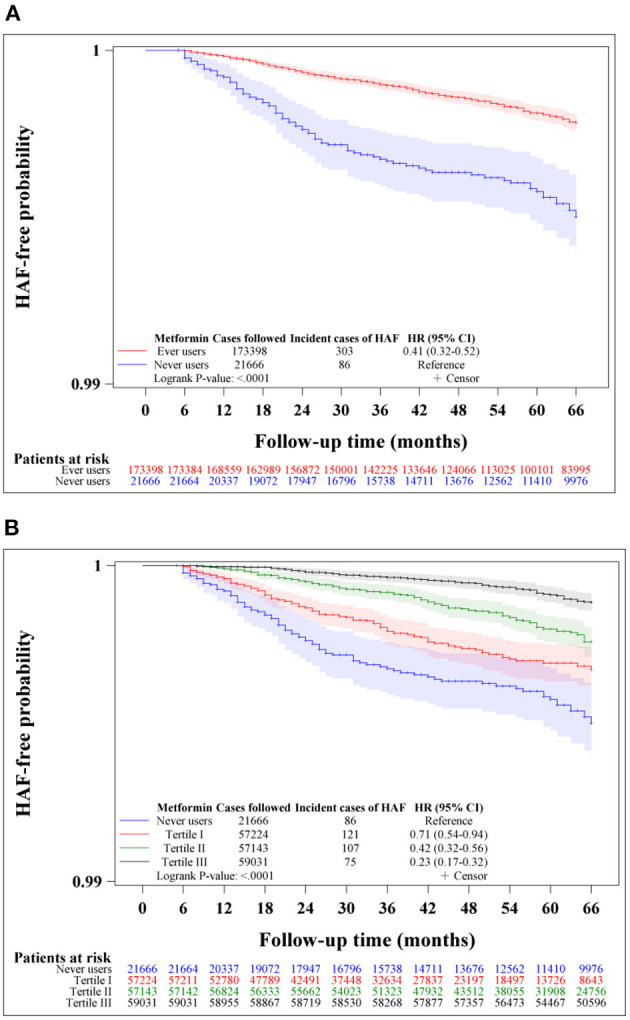
Kaplan-Meier curves comparing hospitalization for atrial fibrillation (HAF)-free probability in never users and ever users of metformin **(A)** and in never users and users in each tertile of cumulative duration of metformin therapy **(B)** in the unmatched cohort. The 95% confidence intervals are shown in shaded areas. HR, hazard ratio; CI, confidence interval.

**Figure 3:**
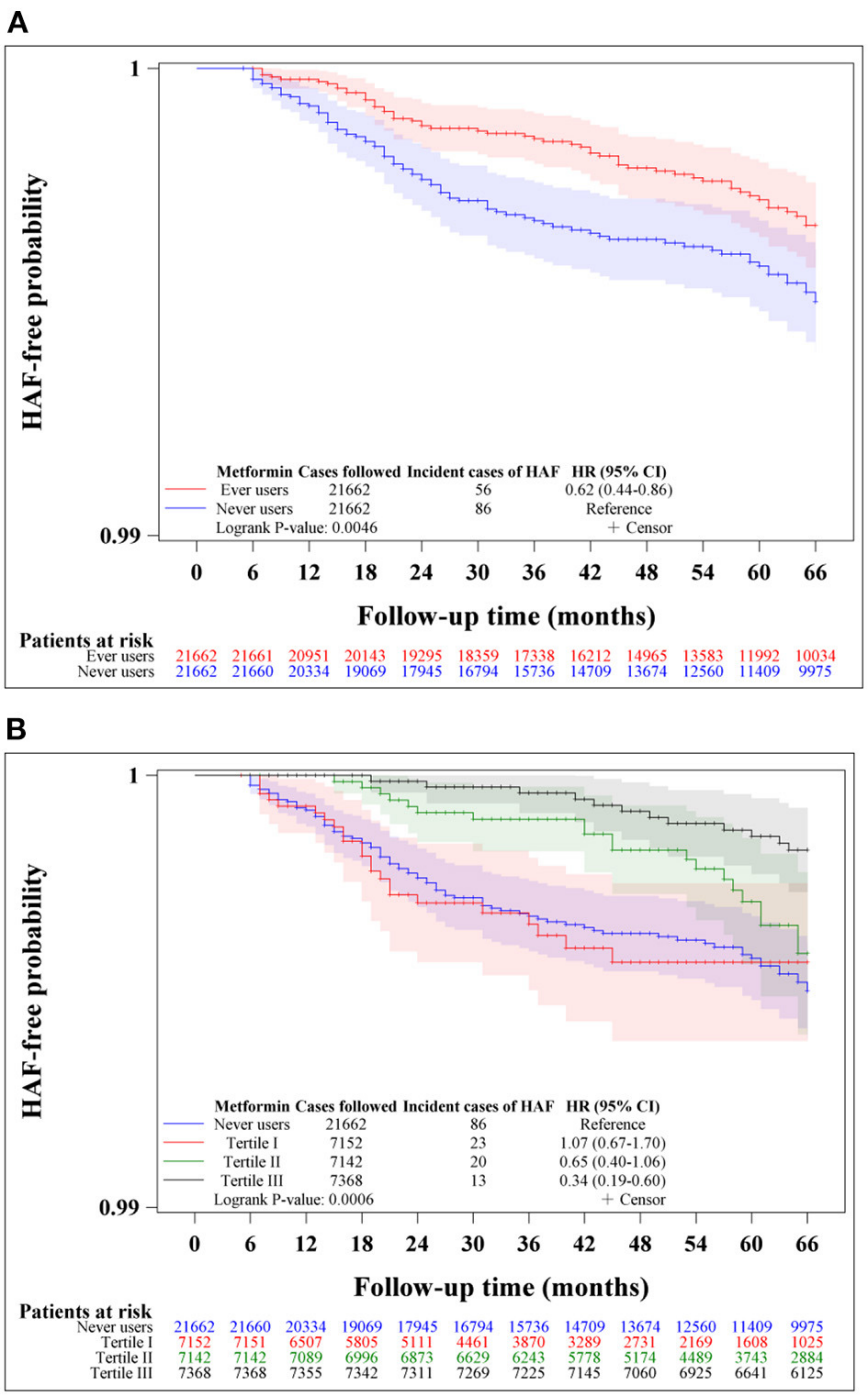
Kaplan-Meier curves comparing hospitalization for atrial fibrillation (HAF)-free probability in never users and ever users of metformin **(A)** and in never users and users in each tertile of cumulative duration of metformin therapy **(B)** in the matched cohort. The 95% confidence intervals are shown in shaded areas. HR, hazard ratio; CI, confidence interval.

Figure 2 shows the Kaplan-Meier curves comparing HAF-free probability in never users and ever users of metformin (Figure 2A) and in never users and users in each of the tertiles of cumulative duration of metformin therapy (Figure 2B) in the unmatched cohort. The logrank test supported significant differences among the various subgroups of metformin exposure. Figure 3 shows the respective curves in the matched cohort and the findings were similar to those observed in the unmatched cohort shown in Figure 2.

Table 2 shows the incidence of HAF and the hazard ratios by metformin exposure in the unmatched cohort and the matched cohort in the main analyses, respectively. A significantly lower risk of HAF in metformin ever users could be demonstrated by the overall hazard ratios and the tertile analyses supported a dose-response relationship.

**Table 2:** Incidence of hospitalization for atrial fibrillation and hazard ratios by metformin exposure in the main analyses.

**Cohort/Metformin use**	***n***	***N***	**Person–year**	**Incidence rate** **(per 100,000 person-years)**	**Hazard ratio**	**95% Confidence interval**	***P* value**
**Unmatched cohort**							
Never users	86	21,666	93021.27	92.45	1.000		
Ever users	303	173,398	803329.81	37.72	0.405	(0.319–0.515)	<0.0001
**Tertiles of cumulative duration of metformin therapy (months)**							
Never users	86	21,666	93021.27	92.45	1.000		
<25.9	121	57,224	194246.52	62.29	0.664	(0.502–0.878)	0.0041
25.9–57.0	107	57,143	274026.03	39.05	0.419	(0.316–0.557)	<0.0001
>57.0	75	59,031	335057.26	22.38	0.230	(0.168–0.314)	<0.0001
**Matched cohort**							
Never users	86	21,662	93008.43	92.46	1.000		
Ever users	56	21,662	98284.01	56.98	0.617	(0.441–0.864)	0.0049
**Tertiles of cumulative duration of metformin therapy (months)**							
Never users	86	21,662	93008.43	92.46	1.000		
<25.3	23	7,152	23486.22	97.93	1.022	(0.643–1.625)	0.9260
25.3–56.5	20	7,142	33396.19	59.89	0.654	(0.402–1.064)	0.0873
>56.5	13	7,368	41401.59	31.40	0.339	(0.189–0.608)	0.0003

*n, incident case number of hospitalization for atrial fibrillation; N, case number followed*.

Table 3 shows the findings in the sensitivity analyses. All analyses supported a lower risk of HAF associated with metformin use. The beneficial effect of metformin was independent of the regularity of metformin refill (Model I), the prescription of incretin-based therapies during follow-up (Model II), the diagnosis of diabetic nephropathy (Models III and IV), and the diagnosis of chronic kidney disease and/or in a renal dialysis status (Models V, VI, and VII).

**Table 3:** Sensitivity analyses.

**Model/metformin use**	***n***	***N***	**Hazard ratio**	**95% Confidence interval**	***P*-value**
**I. Excluding two consecutive prescriptions of metformin spanning more than 4 months**					
Never users	86	21,666	1.000		
Ever users	93	59,513	0.393	(0.293–0.527)	<0.0001
**Tertiles of cumulative duration of metformin therapy (months)**					
Never users	86	21,666	1.000		
<25.2	35	20,075	0.708	(0.474–1.058)	0.0922
25.2–56.4	27	16,197	0.415	(0.269–0.640)	<0.0001
>56.4	31	23,241	0.245	(0.162–0.370)	<0.0001
**II. Excluding patients treated with incretin–based therapies during follow–up**					
Never users	84	20,402	1.000		
Ever users	269	134,549	0.458	(0.359–0.585)	<0.0001
**Tertiles of cumulative duration of metformin therapy (months)**					
Never users	84	20,402	1.000		
<25.2	115	48,775	0.713	(0.536–0.947)	0.0196
25.2–56.4	94	44,083	0.465	(0.347–0.625)	<0.0001
>56.4	60	41,691	0.258	(0.185–0.361)	<0.0001
**III. Patients with nephropathy**					
Never users	46	8,376	1.000		
Ever users	111	50,614	0.347	(0.246–0.490)	<0.0001
**Tertiles of cumulative duration of metformin therapy (months)**					
Never users	46	8,376	1.000		
<25.2	38	17,930	0.454	(0.294–0.700)	0.0004
25.2–56.4	38	16,903	0.343	(0.223–0.527)	<0.0001
>56.4	35	15,781	0.275	(0.177–0.428)	<0.0001
**IV. Patients without nephropathy**					
Never users	40	13,290	1.000		
Ever users	192	122,784	0.494	(0.351–0.694)	<0.0001
**Tertiles of cumulative duration of metformin therapy (months)**					
Never users	40	13,290	1.000		
<25.2	83	39,294	0.895	(0.611–1.311)	0.5704
25.2–56.4	69	40,240	0.525	(0.355–0.775)	0.0012
>56.4	40	43,250	0.227	(0.146–0.353)	<0.0001
**V. Excluding patients with a diagnosis of chronic kidney disease**					
Never users	49	17,254	1.000		
Ever users	246	155,114	0.524	(0.386–0.712)	<0.0001
**Tertiles of cumulative duration of metformin therapy (months)**					
Never users	49	17,254	1.000		
<25.2	101	50,213	0.911	(0.645–1.288)	0.5989
25.2–56.4	86	51,063	0.540	(0.380–0.768)	0.0006
>56.4	59	53,838	0.280	(0.191–0.410)	<0.0001
**VI. Excluding patients in a renal dialysis status**					
Never users	81	21,082	1.000		
Ever users	301	172,642	0.418	(0.327–0.535)	<0.0001
**Tertiles of cumulative duration of metformin therapy (months)**					
Never users	81	21,082	1.000		
<25.2	120	56,839	0.684	(0.515–0.910)	0.0091
25.2–56.4	107	56,865	0.435	(0.326–0.581)	<0.0001
>56.4	74	58,938	0.235	(0.171–0.322)	<0.0001
**VII. Excluding patients with chronic kidney disease and/or in a renal dialysis status**					
Never users	49	17,252	1.000		
Ever users	245	155,099	0.522	(0.384–0.709)	<0.0001
**Tertiles of cumulative duration of metformin therapy (months**)					
Never users	49	17,252	1.000		
<25.2	100	50,206	0.901	(0.638–1.274)	0.5566
25.2–56.4	86	51,057	0.540	(0.380–0.768)	0.0006
>56.4	59	53,836	0.280	(0.191–0.410)	<0.0001

*n, incident case number of hospitalization for atrial fibrillation, N, case number followed*.

## Discussion

This study confirmed a lower risk of HAF in patients with type 2 diabetes mellitus who had been treated with metformin (Tables 2, 3, Figures 2, 3). A dose-response pattern was seen and the beneficial effect was especially remarkable when metformin had been used for more than 2 years in either the main analyses (Table 2) or the sensitivity analyses (Table 3).

While compared with the previous two studies conducted in Taiwan that investigated the effect of metformin on AF (11, 12), the present study has several merits and methodological improvements. The study by Chang et al. showed a high imbalance between metformin users and non-users in the use of statin, the distribution of major risk factors and the use of other antidiabetic drugs (11). All of these have been considered in the present study and the distributions of these potential confounders were well-balanced in our analyses in the matched cohort (Table 1). Furthermore, Chang et al. did not address the potential bias resulting from immortal time (11) and this has been considered in the present study (discussed later). The study by Liou et al. used a cross-sectional design and also had imbalanced distributions of potential confounders and the use of other antidiabetic drugs (12). The present study considered the balance of potential confounders and the use of other antidiabetic drugs in the matched cohort (Table 1) and used a retrospective cohort design that addressed the correct temporality between exposure (metformin use) and effect (HAF).

Additionally, both previous studies did not consider a dose-response effect and the regularity in the prescription of metformin and the investigators included the diagnosis of AF made at the outpatient clinics (11, 12). The present study reinforced the robustness of the preventive effect of metformin on AF by showing a dose-response effect (Tables 2, 3) and a consistency of the findings after excluding patients who had not received regular refill of metformin (Model I of Table 3). The present study defined AF as a primary diagnosis made at hospital discharge which should have been supported by laboratory information such as electrocardiograms or electrophysiological studies for the reimbursement purpose.

It is worthy to note that, by using the diagnostic code mainly identified at the outpatient clinics, the hazard ratio of 0.81 (95% confidence interval 0.76–0.86) estimated by Chang et al. (11) and the odds ratio of 0.81 (95% confidence interval 0.71–0.93) estimated by Liou et al. (12) showed a less magnitude of protection than what we have seen in the present study (Tables 2, 3). This could be because that the diagnostic code of AF labeled at the outpatient clinics was not as accurate as the diagnosis made during hospitalization. The misclassification made at outpatient clinics was probably non-differential and thus biased the hazard ratios toward the null (23). It is true that when we used a less stringent definition of AF by including the diagnostic code identified either at the outpatient clinics or during hospitalization in secondary analyses, the estimated hazard ratios did move toward the null (overall hazard ratio for the unmatched cohort: 0.426, 95% confidence interval: 0.395–0.459; and for the matched cohort: 0.667, 95% confidence interval: 0.601–0.740), indicating a prone to non-differential misclassification by using the diagnosis made at the outpatient clinics. However, despite the variation in the estimated risk ratios, all studies consistently supported a protection of metformin against AF by using different study designs and different statistical analyses and by defining AF either as a diagnosis made at the outpatient clinics or made at the discharge of a hospitalization.

The higher risk of AF in patients with type 2 diabetes mellitus is assumed to be related to metabolic syndrome and the pathogenesis may involve insulin resistance, hypertension, greater glycemic excursion, hypoglycemia, myocardial steatosis, endothelial dysfunction, inflammation, and left atrial dilatation and fibrosis (3). These can lead to electrical and structural remodeling and AF (3). Therefore, even though the mechanisms of a reduced risk of AF associated with metformin use are not yet elucidated, the biological actions of metformin targeting the pathophysiology of the development of AF might have contributed to such a clinical benefit.

Metformin improves insulin resistance by increasing the expression of insulin receptor and activation of tyrosine kinase (24). In the absence of co-administration of insulin or insulin-stimulating drugs, metformin *per se* rarely induce hypoglycemia. On the other hand, microvascular dysfunction and relative ischemia with reduced oxygen/nutrient delivery and/or increased energy demand in the heart may lead to metabolic stress and induce AF (1). Through the activation of 5′-adenosine monophosphate-activated protein kinase, metformin promotes fatty acid oxidation, increases ketone body metabolism, reduces lipid accumulation, and induces the expression of glucose transporter in cardiomyocytes, thus facilitates more efficient energy use with reduction of metabolic stress (1, 25). Studies also suggested that metformin may reduce AF by alleviating the dysfunction of epicardial adipose tissue (26). In an *in vitro* study, metformin reduces the production of reactive oxygen species and myolysis during tachypacing cell culture of atrial myocytes (11).

Pro-fibrotic biomarkers such as interleukin-6, transforming growth factor-beta one, matrix metalloproteinase-9 and tissue inhibitor of metalloproteinase-1 are important biomarkers of atrial remodeling in AF (1, 27). Interestingly, metformin inhibits the signaling pathways of transforming growth factor-beta one (28). Taken together, the mechanisms of a reduced risk of AF associated with metformin use may be multifactorial and are related to a reduction of insulin resistance and metabolic stress, inhibition of inflammation, and alleviation of cardiac fibrosis.

The findings of the present study have some clinical implications. First, because diabetes patients have a higher risk of AF (2–4) which may contribute to the significantly higher risk of thromboembolic events and mortality (5), the reduced risk of AF associated with metformin use provided a good rationale for the recommendation of metformin as the first-line treatment for glucose lowering in patients with type 2 diabetes mellitus. Second, it would be a good strategy to continue the use of metformin when the addition of other antidiabetic drugs is required for better control of hyperglycemia because its protection against AF may or may not be directly related to glucose lowering and such a protection was observed mainly after 2 years of its use (Tables 2, 3). Furthermore, the dose-response pattern (Tables 2, 3) not only implicates a cause-effect relationship but also provides a good rationale for its continuous use when other antidiabetic drugs are added for the improvement of hyperglycemia. Third, the consistent findings of a protective role of metformin on AF in the diabetes patients might provide rationale for more vigorous investigation of its usefulness in non-diabetes people who are at a high risk of developing AF.

Pharmacoepidemiological studies using administrative databases to evaluate long-term safety or beneficial/adverse effects of medications have become popular in recent years. These studies are most useful for outcomes with low incidence or when randomized controlled trials are not feasible. However, some methodology limitations should be carefully addressed for not getting a biased result. Methodological limitations such as selection bias, prevalent user bias, immortal time bias, and confounding by indication as seen in the two previous studies (11, 12) have been carefully addressed in the present study during study design, patient enrollment and statistical analyses. Selection bias was avoided by using the nationwide database covering > 99% of the population and prevalent user bias was prevented by enrolling patients with new-onset diabetes and new users of metformin. The impacts or carryover effects of other antidiabetic drugs used before metformin was initiated were also excluded by enrolling only ever users of metformin who received metformin as the first antidiabetic drug (Figure 1).

Immortal time refers to the follow-up period during which the outcome cannot happen. Immortal time bias can be introduced when treatment status and/or follow-up time are inappropriately assigned (29). To prevent misclassification of non-diabetes people as diabetes patients, those with uncertain diabetes diagnosis have been excluded by enrolling only patients who had been prescribed antidiabetic drugs for two or more times (Figure 1). Misclassification of the treatment status of metformin was not likely because the NHI is a universal health care system and the information of all prescriptions is available during the long period of follow-up. The immortal time during the period between diabetes diagnosis and the initiation of antidiabetic drugs and during the initial short follow-up period of <180 days were not included in the calculation of the follow-up person-years. Lastly, the immortal time during the waiting period between drug prescription and drug dispense when a patient is discharged from the hospital [as pointed out by Lévesque et al. (29)] is not a problem in Taiwan because the patient can get all discharge medications immediately from the hospital at the time of his/her discharge.

There are some additional strengths. First, recall bias resulting from self-reporting would not happen in the study because medical records were used. Second, although the detection rate of a disease might be affected by socioeconomic status of the patients in studies conducted in other countries, this was less likely in Taiwan because the drug cost-sharing in the NHI health care system is low and much expenses can be waived in veterans, in patients with low-income or when the patients receive prescription refills for chronic disease.

The consistency of the findings in both the unmatched cohort and the matched cohort (Table 2) and in the sensitivity analyses (Table 3) suggested that the results were reproducible in different cohorts. The use of a PS-matched cohort (Table 1) and the estimation of hazard ratios by using the Cox proportional hazards model incorporated with IPTW (Tables 2, 3) was aimed at reducing potential confounding by indication. The possibility of residual confounding from the covariates should be small, especially in the matched cohort for whom the values of standardized difference between ever and never users of metformin were < 10% for all covariates (Table 1).

There are some limitations in the present study. First, we did not have electrocardiograms for confirming the diagnosis of AF. Therefore, misclassification of AF could not be completely excluded. However, if the misclassifications were not differential in ever and never users of metformin, the hazard ratios would only have been underestimated (23). Second, blood glucose levels were not available in the database. Because a cause should happen before an effect, the lack of blood glucose levels prevents us from more affirmative assurance of the correctness of temporality between diabetes as a cause and AF as an effect in some patients. Third, in the matched cohort, the additional treatment with metformin in ever users who had balanced use of other antidiabetic drugs while compared to never users of metformin suggested that metformin users might have a higher intensity of medications. This either implied that ever users might have a more severe disease condition or that blood glucose control was not balanced between the two groups. Because ever users and never users of metformin were balanced in all covariates in the matched cohort (Table 1), a discrepancy in disease severity in terms of diabetes complications or comorbidities was less likely. However, a discrepancy in blood glucose control between the two groups could not be ruled out. Because blood glucose fluctuations and insulin resistance may be related to the pathogenesis of AF and the information of these parameters was not available in the database, future studies are required to clarify the role of glycemic control and insulin resistance in the discrepant effects observed in ever users and never users of metformin. Fourth, in the sensitivity analyses, although we have separately conducted subgroup analyses in patients with nephropathy (Model III, Table 3) and without nephropathy (Model IV, Table 3); and after excluding patients with a diagnosis of chronic kidney disease and/or in a renal dialysis status (Models V, VI, and VII, Table 3), we did not have data of urinary albumin excretion rate or estimated glomerular filtration rate for more accurate diagnosis. Fifth, obesity and tobacco use might have been underestimated by using the diagnostic codes because these diagnoses were mostly not directly related to reimbursement purpose. However, because clinical diseases related to obesity and tobacco use such as hypertension, dyslipidemia, chronic obstructive pulmonary disease, cancer, and cardiovascular diseases have also been considered as potential confounders in the analyses (Table 1), it is believed that their effects on the estimation of hazard ratios might also have been adjusted for. Sixth, knowledge of absolute risk reduction and number needed to treat may be of clinical importance (30). As the incidence of HAF was low, the absolute risk reduction calculated from the matched cohort was too small (86/21,662–56/21,662 = 0.14%) and the calculated number needed to treat was too large (the reciprocal of absolute risk reduction = 722). Therefore, the cost effectiveness of using metformin to prevent HAF remains to be investigated. Seventh, because this is a retrospective cohort study, the findings should better be confirmed by prospective cohort study designs or by clinical trials. Finally, we did not have measurement data of some other confounders like biochemical and hormonal data, anthropometric factors, lifestyle, physical activity, dietary pattern, cigarette smoking, alcohol drinking, family history, and genetic parameters.

## Conclusion

This study supports a lower risk of HAF in patients with type 2 diabetes mellitus who have been treated with metformin. However, additional prospective observational studies and/or clinical trials are necessary to confirm a cause-effect relationship. Because metformin is inexpensive and safe and does not cause hypoglycemia when it is used as a monotherapy, the usefulness of metformin as a protection against AF in high risk patients is worthy of more intensive investigation in both the diabetes patients and the non-diabetes people.

## Data Availability Statement

The datasets presented in this article are not readily available because public availability of the dataset is restricted by local regulations to protect privacy. Requests to access the datasets should be directed to Chin-Hsiao Tseng, ccktsh@ms6.hinet.net.

## Ethics Statement

The studies involving human participants were reviewed and approved by National Health Research Institutes. Written informed consent from the participants' legal guardian/next of kin was not required to participate in this study in accordance with the national legislation and the institutional requirements.

## Author Contributions

C-HT researched data and wrote manuscript.

## Conflict of Interest

The author declares that the research was conducted in the absence of any commercial or financial relationships that could be construed as a potential conflict of interest.

## Abbreviations

AF: atrial fibrillation
HAF: hospitalization for atrial fibrillation
ICD-9-CM, International Classification of Diseases, Ninth Revision: Clinical Modification
IPTW: inverse probability of treatment weighting
NHI: National Health Insurance
PS: propensity score.
